# The β-Hemolysin and Intracellular Survival of *Streptococcus agalactiae* in Human Macrophages

**DOI:** 10.1371/journal.pone.0060160

**Published:** 2013-04-04

**Authors:** Anubha Sagar, Carolin Klemm, Lara Hartjes, Stefanie Mauerer, Ger van Zandbergen, Barbara Spellerberg

**Affiliations:** 1 Institute of Medical Microbiology and Hospital Hygiene, University of Ulm, Ulm, Germany; 2 Federal Institute of Vaccines and Bio-Medical Drug, Paul-Ehrlich-Institute, Langen, Germany; Columbia University, United States of America

## Abstract

*S. agalactiae* (group B streptococci, GBS) is a major microbial pathogen in human neonates and causes invasive infections in pregnant women and immunocompromised individuals. The *S. agalactiae* β-hemolysin is regarded as an important virulence factor for the development of invasive disease. To examine the role of β-hemolysin in the interaction with professional phagocytes, the THP-1 monocytic cell line and human granulocytes were infected with a serotype Ia *S. agalactiae* wild type strain and its isogenic nonhemolytic mutant. We could show that the nonhemolytic mutants were able to survive in significantly higher numbers than the hemolytic wild type strain, in THP-1 macrophage-like cells and in assays with human granulocytes. Intracellular bacterial multiplication, however, could not be observed. The hemolytic wild type strain stimulated a significantly higher release of Tumor Necrosis Factor-α than the nonhemolytic mutant in THP-1 cells, while similar levels of the chemokine Interleukin-8 were induced. In order to investigate bacterial mediators of IL-8 release in this setting, purified cell wall preparations from both strains were tested and found to exert a potent proinflammatory stimulus on THP-1 cells. In conclusion, our results indicate that the β-hemolysin has a strong influence on the intracellular survival of *S. agalactiae* and that a tightly controlled regulation of β-hemolysin expression is required for the successful establishment of *S. agalactiae* in different host niches.

## Introduction

The Gram positive pathogen *Streptococcus agalactiae* or group B streptococcus (GBS) is the leading microbial agent of neonatal pneumonia, sepsis and meningitis presenting as early or late-onset disease in human newborns [1] [2]. In most cases, vertical transmission of *S. agalactiae* to the neonate occurs during delivery from colonized mothers. But bacteria can also be acquired through horizontal transfer from external sources, for example, in the intensive care unit of the hospital [3]. A rising incidence of invasive infections in recent years has been described for nonpregnant adults as well as elderly and immunocompromised populations [4].

The *S. agalactiae* β-hemolysin is regarded as an important virulence factor for the development of invasive disease. Several studies have determined the role of the *S. agalactiae* surface-associated β-hemolysin as a membrane destabilizing toxin in lung epithelial cells [5], and brain endothelial cells [6] which contributes to disease pathogenesis. However, controversial reports exist regarding the role of β-hemolysin for the survival of *S. agalactiae* in phagocytes. Liu *et al.* found that a deletion in the *cylE* gene renders the pathogen sensitive to host phagocytic clearance mechanisms [7]. On the other hand, Sendi *et al*. [8] have shown the critical involvement of the *S. agalactiae* CovR/S (also called CsrR/S) [9] two-component global regulatory system in virulence. Mutations in the *cov* gene lead to an increased β-hemolytic activity with lower capsule expression. The resultant phenotype showed impaired intracellular survival in neutrophils in contrast to the LH/HC phenotype. Nevertheless, which role the β-hemolysin plays for the observed effect remains to be determined.

The *cyl* locus of *Streptococcus agalactiae* encodes β-hemolysin activity and consists of a cluster of genes. While a typical ABC transporter for the extrusion of the β-hemolysin is present in this gene cluster, the other genes appear to encode proteins with similarities to fatty acid synthesis enzymes [10] [11]. In this study, we have used a wild type strain and an isogenic mutant deficient in *cylA* that encodes the ATP- binding domain of the β-hemolysin transporter [11]. We investigated the role of the β-hemolysin for survival of *S. agalactiae* within THP-1 monocytic cells and primary human granulocytes as relevant host cells of the innate immune system.

## Materials and Methods

### Streptococcal Strains and Growth Conditions

The bacterial strains and plasmids used are listed in Table 1. BSU 6 a *S. agalactiae* serotype Ia strain served as the wild type. The isogenic β-hemolysin deletion mutant of this strain, designated as BSU 281, was generated by deleting the *cylA* gene that encodes the ATP-binding domain of the β-hemolysin transporter as described in an earlier publication [11]. For immunofluorescence microscopy, both streptococcal strains were transformed with the reporter plasmid pBSU101 as described previously [12]. The plasmid carries a copy of the enhanced green fluorescent gene *egfp* under the control of the *S. agalactiae* CAMP- factor gene (*cfb*) promoter. The bacterial strains carrying pBSU101 were designated as BSU 98 (parent strain: BSU 6) and BSU 453 (parent strain: BSU 281).

**Table 1:** Bacterial strains and plasmids.

*S. agalactiae* strains	Description	Source or reference
BSU 6 serotype Ia strain	Clinical isolate; Hly^+^	Ulm collection
BSU 281	BSU 6 derivative; *ΔcylA;* Hly^−^	Gottschalk et al., 2006 [11]
BSU 98	BSU 6 derivative carrying plasmid pBSU101; Hly^+^	This study
BSU 453	BSU 281 derivative carrying plasmid pBSU101; Hly^−^	This study
**Plasmids**		
pAT28	Spec^r^; ori pUC; ori pAMβ1	Trieu-Cuot et al., 1990 [39]
pBSU101	pAT28 derivative carrying *egfp* under the control of the *cfb* promoter	Aymanns et al., 2011 [12]

Hly^+^ refers to hemolytic isolates.

Hly^−^refers to nonhemolytic isolates.

The bacteria were grown at 37°C in Todd-Hewitt broth (THB; Oxoid, Wesel, Germany) supplemented with 0.5% yeast extract (Difco) containing 120 mg/l Spectinomycin. For experiments, bacteria were adjusted to 10^7^ colony forming units (CFU)/ml, washed in phosphate-buffered saline (PBS, pH 7.0) and resuspended in RPMI-1640 cell culture medium (Sigma-Aldrich, Deisenhofen, Germany).

### THP-1 Cells as a Model for Human Macrophages

THP-1 (ATCC, East Greenwich, RI, USA) is a human acute monocytic leukemia cell line. Morphologically they appear as large, round single, non adherent cells. They were grown at a density of 2×10^5^ cells/ml in RPMI1640 medium supplemented with 10% γ-irradiated FBS, 50 µM β-mercaptoethanol, 2 mM L-Glutamine, 100 U/100 µg/ml Penicillin/Streptomycin, 2 mM HEPES (all from Biochrom, Berlin, Germany). Cells were maintained in a humidified atmosphere with 5% CO_2_ at 37°C.

Cells were passaged when density reached 8×10^5^ cells/ml and media were changed every three days. For experiments, 10^6^ cells were seeded into each well of a 6-well tissue culture plates (Becton Dickinson) and kept in the presence of 10 ng/ml Phorbol 12-myristate 13-acetate (PMA, Sigma, Deisenhofen, Germany) overnight at 37°C and 5% CO_2_ [13]. On the next day, THP-1 monocytes were completely differentiated into adherent macrophages; a phenomenon controlled via light microscopy. THP-1 macrophages were washed three times with RPMI 1640 medium without antibiotics followed by adding the same medium for the rest of the infection assays.

### Isolation of Human Peripheral Blood Granulocytes

Peripheral blood was collected by venipuncture from healthy adult volunteers who gave informed written consent to donate blood specifically for the purpose of the study. The ethics committee at the University of Ulm approved this procedure. Granulocytes were isolated as described elsewhere [14]. Briefly, a two-layer density gradient consisting of a bottom layer of Histopaque 1119 (Sigma-Aldrich, Deisenhofen, Germany) and an upper layer of lymphocyte separation medium 1077 (PAA, Pasching, Austria) was prepared. Blood was layered carefully on top and centrifuged for 5 min at 1000 rpm, followed by 25 min at 2200 rpm at room temperature. The pink layer at the interface between the two gradients, which is formed mainly by granulocytes, was collected and washed with 1×PBS (PAA, Austria) and resuspended in RPMI 1640 medium. The cells were further fractioned on a discontinuous Percoll (Amersham Biosciences, Uppsala, Sweden) gradient consisting of layers with densities of 1.105 g/ml (85%), 1.100 g/ml (80%), 1.087 g/ml (70%), and 1.081 g/ml (65%) at 2200 rpm for 25 min. Purified granulocytes forming approximately 2–3 interfaces between 70% to 80% of Percoll layers were collected. The cells were washed with PBS and resuspended in RPMI 1640 medium supplemented with 10% γ-irradiated FBS. The cell preparations contained mostly granulocytes as determined by morphological examination of the cells after Giemsa-staining. Trypan blue exclusion confirmed >99% viability of cells by this procedure.

### Intracellular *S. agalactiae* Survival Assay in THP-1 Macrophages

In order to quantify the intracellular bacteria, 10^6^ THP-1 macrophages per well were infected with the hemolytic (BSU 98) and nonhemolytic (BSU 453) strain at a multiplicity of infection (MOI) of 1∶1 for 0.75 and 1.5 h. Extracellular bacteria were killed using 1 µg/ml Penicillin G and 100 µg/ml Gentamicin (both from Sigma-Aldrich) for 1 h. Subsequently, the medium was removed from each well and cold distilled water was added to lyse the cells with repeated pipetting. The lysates were plated, in various dilutions, on THY agar plates (containing 120 mg/l Spectinomycin) and incubated overnight at 37°C with 5% CO_2._ Colony counts were performed to determine the number of intracellular bacteria.

To assess the effect of inhibitors of the eukaryotic cytoskeleton, assays were performed in the presence of Cytochalasin D (Sigma) at final concentrations of 0.5, 1, 2.5 and 5 µg/ml. Cytochalasin D was added to each well 30 min before infection and was present during the entire experiment.

### *S. agalactiae* Survival Assay in Human Granulocytes

Infection of 10^5^ freshly isolated granulocytes with hemolytic and non hemolytic *S. agalactiae* strains was carried out at a MOI of 1∶1 and incubated at 37°C with 5% CO_2_ for 2 h. In order to determine the total number of viable bacteria, eukaryotic cells were collected by centrifugation at 4000 rpm for 10 min. The pellet was resuspended in 5 ml of ice cold distilled water to lyse granulocytes. The lysate was plated, in various dilutions, on THY agar plates (containing 120 mg/l Spectinomycin) and incubated overnight at 37°C with 5% CO_2._ Colony counts were performed to determine the total number of viable bacteria.

### Lactate Dehydrogenase (LDH) Cytotoxicity Assay

Bacterial cell-mediated cytotoxicity to THP-1 macrophages and granulocytes was detected by measuring LDH activity present in the culture supernatant. For LDH measurements, the Cytotoxicity detection kit (Clontech, USA) was used according to the manufacturer’s instructions. The amount of enzyme was determined in a microplate reader (absorbance at 492 nm, reference wavelength at 620 nm).

### Visualizing Intracellular *S. agalactiae* by Fluorescence Microscopy

0.5×10^6^ THP-1 cells were seeded in each well of a 12-well tissue culture plate. In the presence of 10 ng/ml PMA, overnight cultures of monocytic cells differentiate into macrophages and become adherent on coverslips present at the bottom of the well. On the next day, the cells were washed three times with RPMI 1640 medium without antibiotics followed by adding the same medium for the rest of the assay. The macrophages were infected with the hemolytic (BSU 98) and nonhemolytic (BSU 453) strain at a MOI 1∶1 and incubated at 37°C with 5% CO_2_ for 1.5 h. The cells were washed three times with 1×PBS followed by fixation with 4% formaldehyde for 20 min. After washing with PBS, the coverslips were air-dried. The cells were stained with Evans Blue (bioMérieux, France) for 30 min in the dark. After washing with PBS, the coverslips were air-dried again in the dark. To mount the cells on a microscopic glass slide, VECTASHIELD mounting medium with DAPI (Vector Laboratories, CA) was used. In this way, the slides can be stored at 4°C in the dark for weeks. Intracellular bacteria within THP-1 macrophages were analyzed by fluorescence microscopy with a Zeiss Axioskop-2® fluorescence microscope fitted with a Axiocam HR camera and Axiovison software version 4.8.

### Intracellular *S. agalactiae* Multiplication Assay in THP-1 Macrophages

10^6^ THP-1 macrophages per well were infected with the hemolytic (BSU 98) and non-hemolytic (BSU 453) strain at a MOI of 1∶1 for 1.5 h. Both Penicillin G (1 µg/ml) and Gentamicin (100 µg/ml) were added to each well and incubated for 1, 2, 3, 4, 5, and 24 h at 37°C with 5% CO_2_ to allow *S. agalactiae* multiplication. The medium was removed from each well and cold distilled water was added to lyse the cells with repeated pipetting. The lysates were plated on THY Agar plates (containing 120 mg/l Spectinomycin) to determine the number of intracellular bacteria.

### Cell Wall Preparations

Purified cell walls were prepared as described elsewhere [15]. Briefly, *S. agalactiae* strains BSU 6 and BSU 281 were grown in THY broth (Todd-Hewitt broth supplemented with 0.5% yeast extract) until mid-logarithmic phase. Bacteria were harvested by centrifugation at 10000 rpm for 10 min at 4°C. At this stage, the pellet can be stored at −20°C. The bacterial pellets were resuspended in cold 50 mM tris-HCl (pH = 7.0) and boiled with sodium dodecyl sulfate (SDS) for 15 min. The resulting denatured bacterial suspensions were then mixed with acid-washed glass beads (150–212 µm, Sigma-Aldrich) and subjected to mechanical breaking (8–10 times in a ribolyser at 14000 rpm for 10 min). The supernatants from all the ribolyser tubes were pooled together and the crude cell wall fragments were obtained by centrifugation at 15000 rpm for 15 min at 4°C. Further purification was achieved by treating the cell wall pellet with DNase (10 mg/ml, Sigma-Aldrich), RNase (50 mg/ml, Sigma-Aldrich) and 20 mM MgSO_4_ for 2 h at 37°C followed by adding 10 mM CaCl_2_ and trypsin (100 µg/ml) and kept for overnight shaking at 37°C. After centrifugation, the cell wall pellet was incubated first with 8 M LiCl followed by 100 mM EDTA for 15 min each at 37°C and then washed at each step with sterile distilled water. A last short centrifugation step in the presence of acetone was performed and the pellet was washed with sterile distilled water. Finally, the suspension containing the cell wall was lyophilized and stored at −20°C for future use. Lyophilized cell wall appears as a white cotton like substance.

For stimulation experiments, lyophilized cell wall was weighed and resuspended to 1 mg/ml in RPMI 1640 medium.

### TNF-α and IL-8 Assays

For cytokine induction assays, 10^6^ THP-1 macrophages per well were infected with the hemolytic (BSU 98) and nonhemolytic strain (BSU 453) at a MOI of 1∶1 for 0, 0.75, 1.5, and 3 h. Following the indicated incubation times, extracellular bacteria were killed using Penicillin G (1 µg/ml) and Gentamicin (100 µg/ml) for 1 h. The cell culture supernatants were collected; cells were removed by centrifugation and stored at −20°C until measurement. Uninfected THP-1 macrophages in medium, bacterial cells in medium without macrophages and medium alone served as controls. Release of Tumor Necrosis Factor-α (TNF-α) and Interleukin-8 (IL-8) was measured by a sandwich-ELISA method using Human IL-8 Cytoset™ (Invitrogen) and Human TNF-α Cytoset™ (Invitrogen).

Additionally, 10^6^ THP-1 macrophages were challenged with various concentrations of *S. agalactiae* cell wall preparations and incubated for 2.45, 6, and 18 h for assays of both cytokines at 37°C with 5% CO_2._ Cell free supernatants were stored at −20°C until cytokine assays were performed.

### Statistical Analysis

The data were analyzed using SPSS18.0 software. The Mann-Whitney U test was performed for at least three independent samples for statistical analysis. Data are expressed as average ± SD. Data were considered significant for p-values <0.05.

## Results

### Effect of β-hemolysin on Survival of *S. agalactiae* in Professional Phagocytes

**Figure 1:**
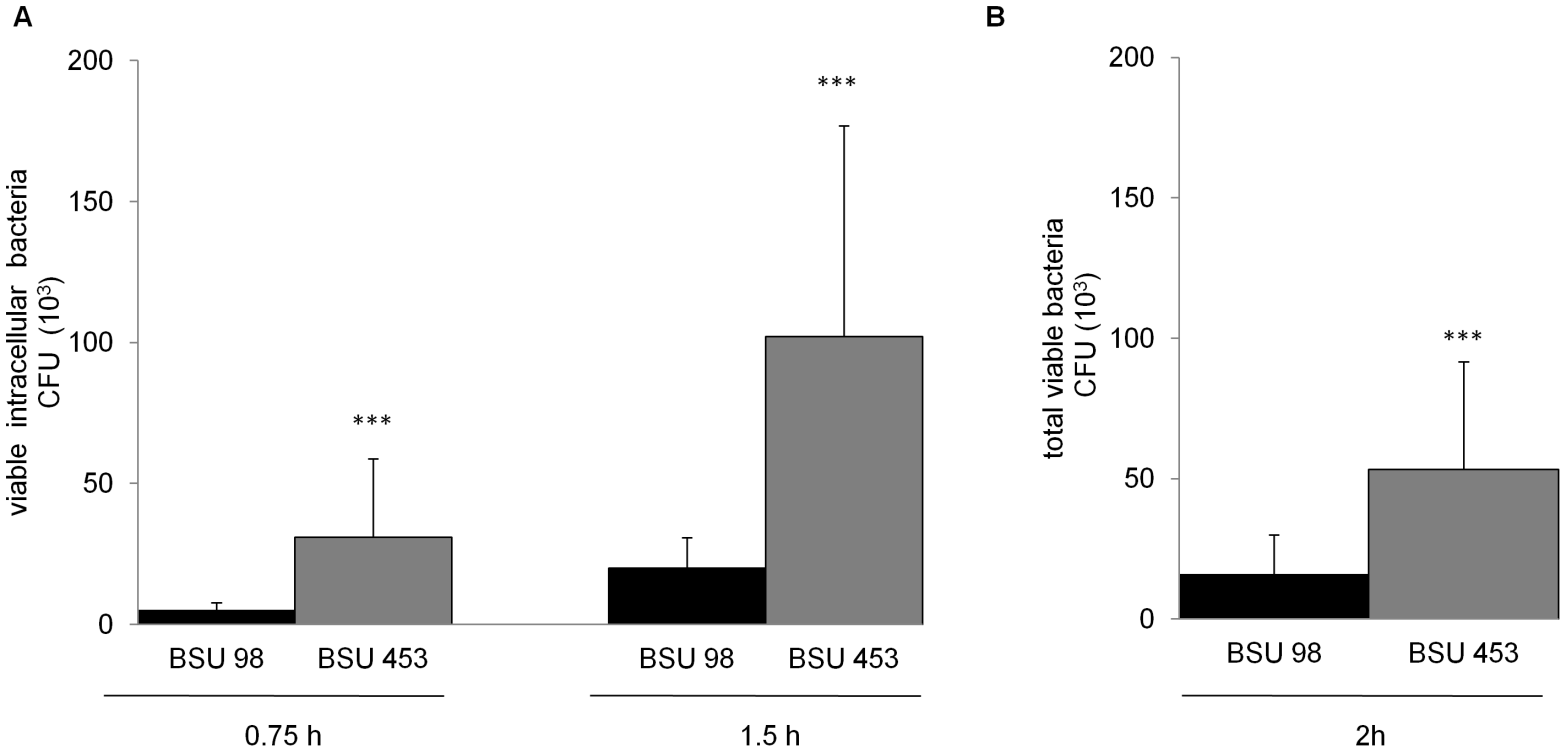
Survival of *S. agalactiae* and β-hemolysin expression in professional phagocytes. The monocyte-derived macrophage cell line THP-1 or freshly isolated granulocytes were infected with hemolytic (BSU 98) and nonhemolytic (BSU 453) bacteria at a MOI of 1∶1 for indicated time points. **A**) Intracellular bacteria were quantified after killing the extracellular bacteria using Penicillin (1 µg/ml) and Gentamicin (100 µg/ml) for additional 1 h. **B**) Total viable bacteria after incubation with granulocytes without killing of extracellular bacteria. Data shown are the mean ± SD of six independent experiments. Data is considered extremely significant for p values <0.001 (***).

A previous study from Sendi *et al.* showed that the hyperhemolytic *S. agalactiae* phenotype (with low capsule expression) displayed impaired survival within human neutrophils as compared to the low hemolytic *S. agalactiae* phenotype (with high capsule expression) [8]. To elucidate the role of the β-hemolysin in this context, we investigated the survival of a hemolytic wild type strain and an isogenic nonhemolytic *S. agalactiae* mutant in phagocytic cells. Intracellular bacterial counts and total survival of the *S. agalactiae* hemolytic wild type (BSU 98) and the nonhemolytic (BSU 453) mutant strain within THP-1 macrophages and human granulocytes were quantified by CFU determination respectively. After infection with the hemolytic wild type strain,the number of intracellular bacteria recovered from THP-1 macrophages was significantly lower in comparison to the nonhemolytic mutant (for 0.75 h and 1.5 h of infection) (Fig. 1A). An MOI of 1 was chosen to ensure sub-cytolytic conditions for the hemolytic as well as the nonhemolytic strain. Similar results were obtained for higher MOI (5 and 10) but under these conditions, increased lysis was observed in THP-1 cells following the incubation with the hemolytic strain BSU 98 (data not shown). A significant difference in survival of the two *S. agalactiae* strains was also observed when incubated with human granulocytes for 2 h without extracellular bacterial killing by antibiotics (Fig. 1B). The hemolytic wild type bacteria display impaired survival in the presence of professional phagocytes as compared to a nonhemolytic *S. agalactiae* mutant strain.

### Cytotoxicity of β-hemolysin on Eukaryotic Host Cells

**Figure 2:**
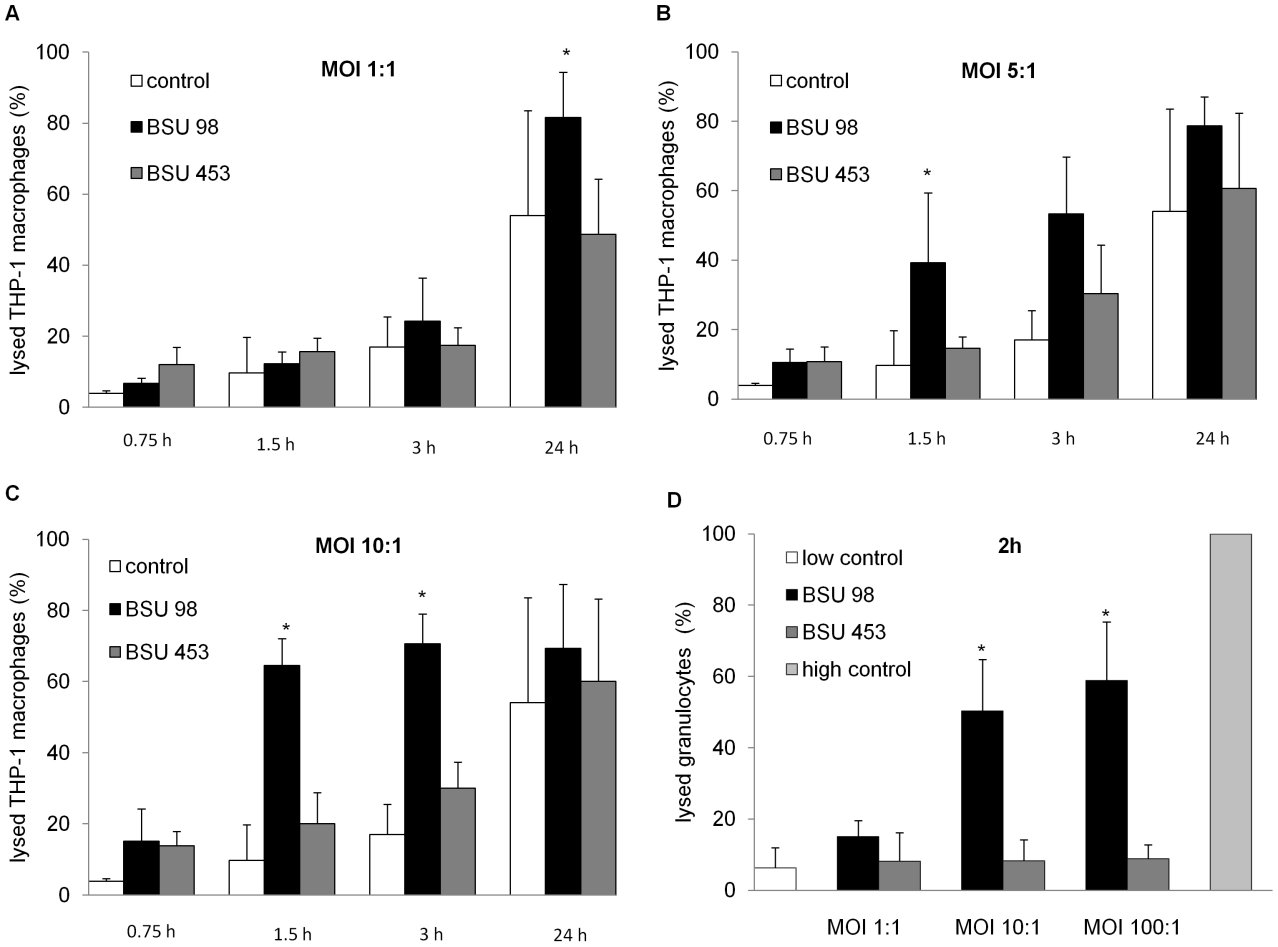
Effect of bacterial cell mediated cytotoxicity as measured by LDH Cytotoxicity Assay. **A–C**) THP-1 macrophages were infected at indicated multiplicity of infections and time points to measure the LDH release into the supernatant. The amount of LDH released is proportional to the percentage of lysed eukaryotic cells. **D**) Human granulocytes were infected at indicated multiplicity of infections for 2 h to measure the LDH release into the supernatant. High control corresponds to maximum lysis achieved using 2% of Triton X-100. Uninfected cells served as control. Data shown are the mean ± SD of three independent experiments. Data is considered significant for p values <0.05 (*).

Since the group B streptococcal β-hemolysin is associated with injury of various eukaryotic cell types including macrophages [5] [16] we quantified the cytotoxic effect of the β-hemolysin on macrophages and granulocytes. We hypothesized that the enhanced lysis of eukaryotic cells infected with the hemolytic strain (BSU 98) could decrease the number of recovered bacteria in comparison to eukaryotic cells infected with the nonhemolytic strain (BSU 453). Lactate Dehydrogenase (LDH) assays were carried out with THP-1 macrophages at MOI of 1, 5 and 10 for 0.75, 1.5, 3 and 24 h. MOI 1, 10 and 100 were tested using human granulocytes for 2 h. The bacterial cell-mediated cytotoxicity can be measured as the amount of the intracellular enzyme LDH released into the culture supernatant by the damaged cells. The LDH release can be directly correlated with the percentage of lysed eukaryotic cells. The results show that at an MOI of 1, strain BSU 98 produced no significant injury to macrophages till 1.5 h, similar to BSU 453 (Fig. 2A). At higher MOIs (5 and 10) the hemolytic strain BSU 98 induced a significant lysis of macrophages compared to BSU 453 (Fig. 2B and 2C). Coincubation of granulocytes with strains BSU 98 and BSU 453 for 2 hours induces basal level of cytotoxicity at an MOI of 1. Higher MOIs (10 and 100) resulted in a more than five-fold increase in the percentage of granulocytes lysed by BSU 98 as compared to the nonhemolytic strain BSU 453 (Fig. 2D).

### Effect of Cytochalasin D on Bacterial Uptake by Macrophages

**Figure 3:**
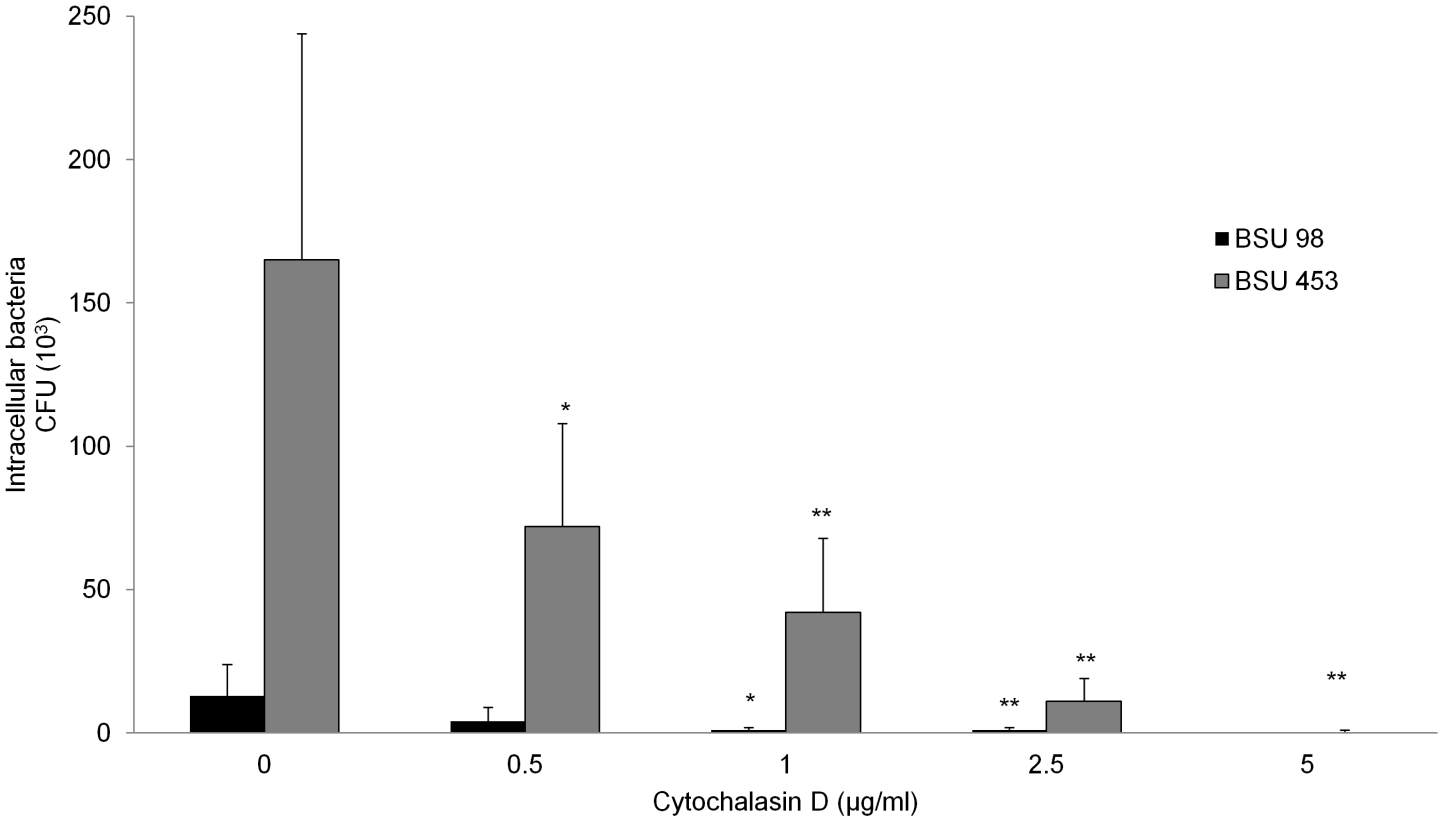
Effect of Cytochalasin D on invasion capacity of *S. agalactiae* in macrophages. Cytochalasin D treated THP-1 macrophages were infected with hemolytic (BSU 98) and nonhemolytic (BSU 453) bacteria for 90 min followed by adding antibiotics for 1 h to kill extracellular bacteria. Infected THP-1 macrophages without Cytochalasin D treatment served as positive control. Data shown are the mean ± SD of six independent experiments. Data is considered significant for p values <0.05 (*) and highly significant for p values <0.01 (**).

Being an actin depolymerizing agent, Cytochalasin D can inhibit actin dependent uptake of *S. agalactiae* by macrophages. To investigate if Cytochalasin D reduces the internalization of the nonhemolytic strain BSU 453 by THP-1 cells to the levels observed for the wild type strain BSU 98, Cytochalasin D was used in a range from 0.5–5 µg/ml. In Fig. 3, Cytochalasin D inhibits the uptake of bacteria and therefore CFU of both strains decreased in a dose-dependent manner. As observed in the previous assays, a significant difference in intracellular colony counts of BSU 98 and BSU 453 is still visible at 0.5 and 1 µg/ml of Cytochalasin D. However, at 5 µg/ml, Cytochalasin D completely inhibited the uptake of both *S. agalactiae* strains, confirming that the number of internalized bacteria in this assay is dependent on the uptake of bacteria into the intracellular compartment.

### Microscopic Evaluation of Intracellular *S. agalactiae* Localization

Using a Zeiss Axioskop-2® fluorescence microscope we visualized the intracellular presence of *S. agalactiae* in eukaryotic cells. To document the subcellular localization of the bacteria within THP-1 macrophages, series of images were acquired from a specimen at equally spaced focus points by moving it along the Z-axis of the microscope. This was combined with three channel fluorescence (blue, green and red) to obtain the resultant Z-stack multichannel images. Distance between the z-planes was set to 1 µm.

**Figure 4:**
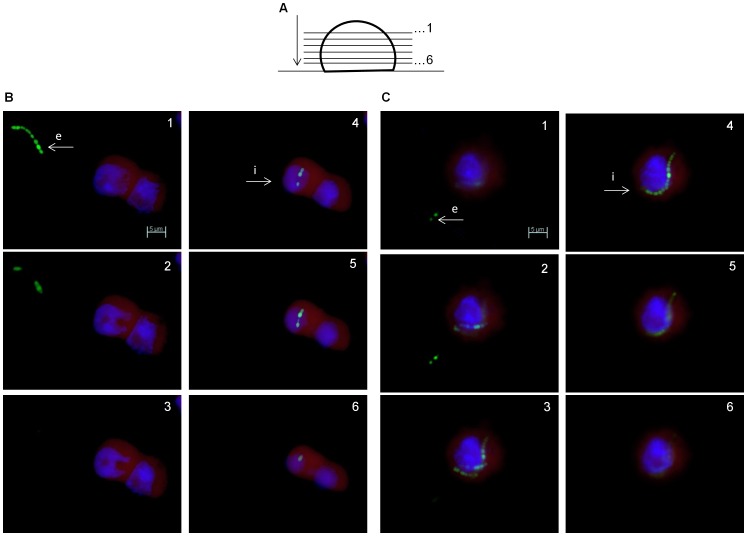
Microscopic evaluation of intracellular *S. agalactiae* localization. **A**) A schematic representation of Z-stacking in Zeiss Axioskop-2® fluorescence microscope. As depicted, images are acquired for six z-stacks through macrophages on a microscopic glass slide with a 63× objective. THP-1 macrophages are infected with BSU 98 (**B**) and BSU 453 (**C**) at a MOI of 1∶1 for 1.5 h. “e” and “i” refer to extracellular and intracellular bacteria respectively. Nuclear staining with DAPI (blue), both *S. agalactiae* strains are EGFP labeled (green) and cytoplasm staining of macrophages with Evans blue (red). Scale Bar: 5 µm (for all images).

As depicted in Fig. 4B and 4C, image series of an infected THP-1 macrophage clearly demonstrate that some bacteria (marked i) are intracellular, whereas others (marked e) are extracellular. On the basis of qualitative comparison, analysis of the infected cells illustrate that within THP-1 macrophages multiple chains of the nonhemolytic bacteria were found more often than in macrophages infected with the hemolytic strain.

### Intracellular *S. agalactiae* Multiplication

**Figure 5:**
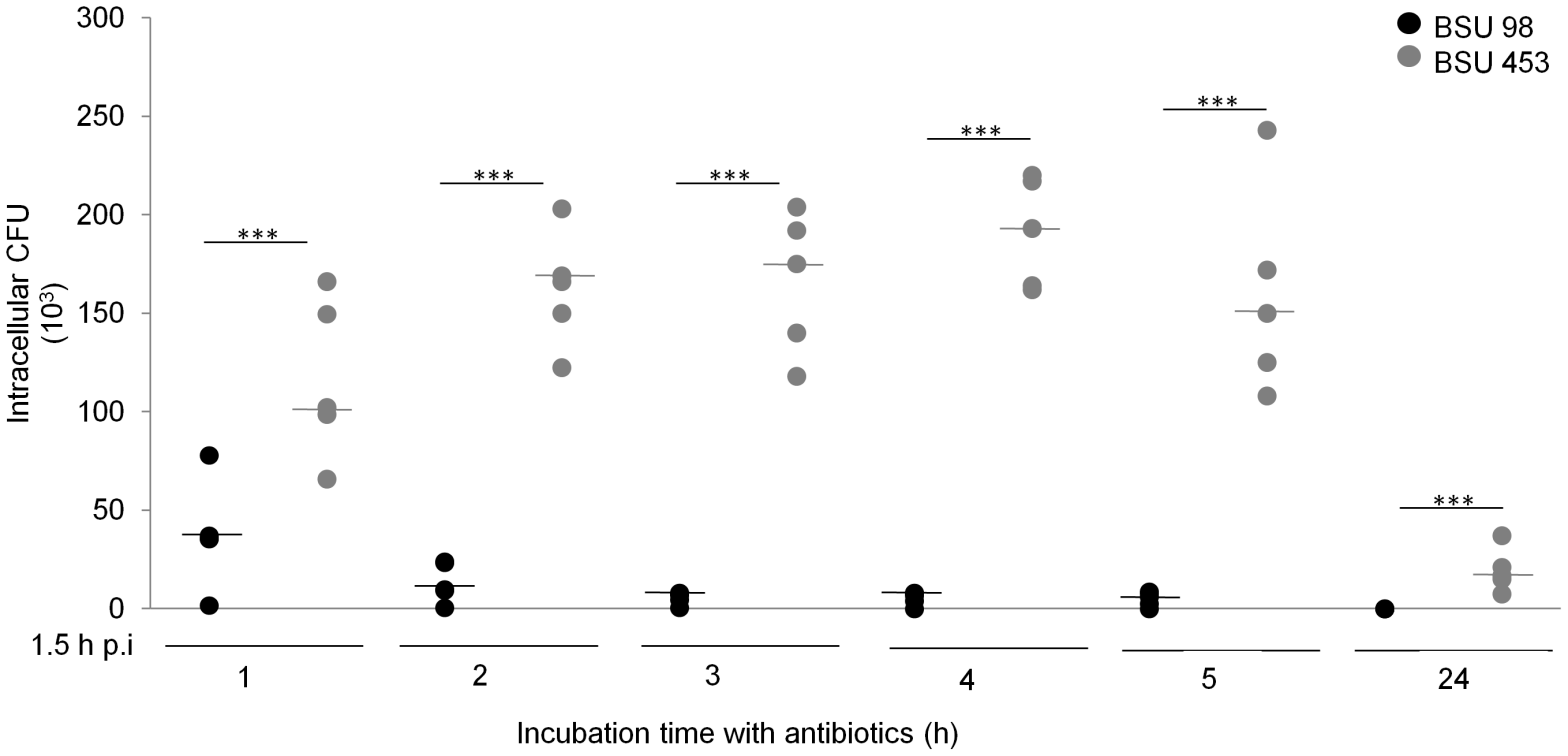
*S. agalactiae* does not multiply within macrophages. THP-1 macrophages were infected with hemolytic (BSU 98) and nonhemolytic (BSU 453) bacteria for 90 min. After this, antibiotics were added into the medium for the rest of the assay. Macrophages were lysed at indicated time points and lysate were plated. Data shown are the values from five independent experiments with median. Data is considered extremely significant for p values <0.001 (***).

Previous literature showed that hemolytic *S. agalactiae* strains do not multiply within the eukaryotic host cell [17]. To analyze if the higher colony counts of *S. agalactiae* strain BSU 453 in our assays were caused by intracellular multiplication of the nonhemolytic mutant, we tested the ability of both strains to multiply within the THP-1 macrophages. As shown in Fig. 5, no significant increase in intracellular CFU was observed between 1 and 5 h of infection. At 24 h, no viable bacteria were recovered, indicating that both *S. agalactiae* strains did not multiply and are eventually killed by the macrophages. These data confirm the enhanced intracellular bacterial counts of BSU 453 in human macrophages, without evidence of a significant intracellular multiplication within phagocytes.

### Cytokine Induction by Type Ia Group B Streptococci

**Figure 6:**
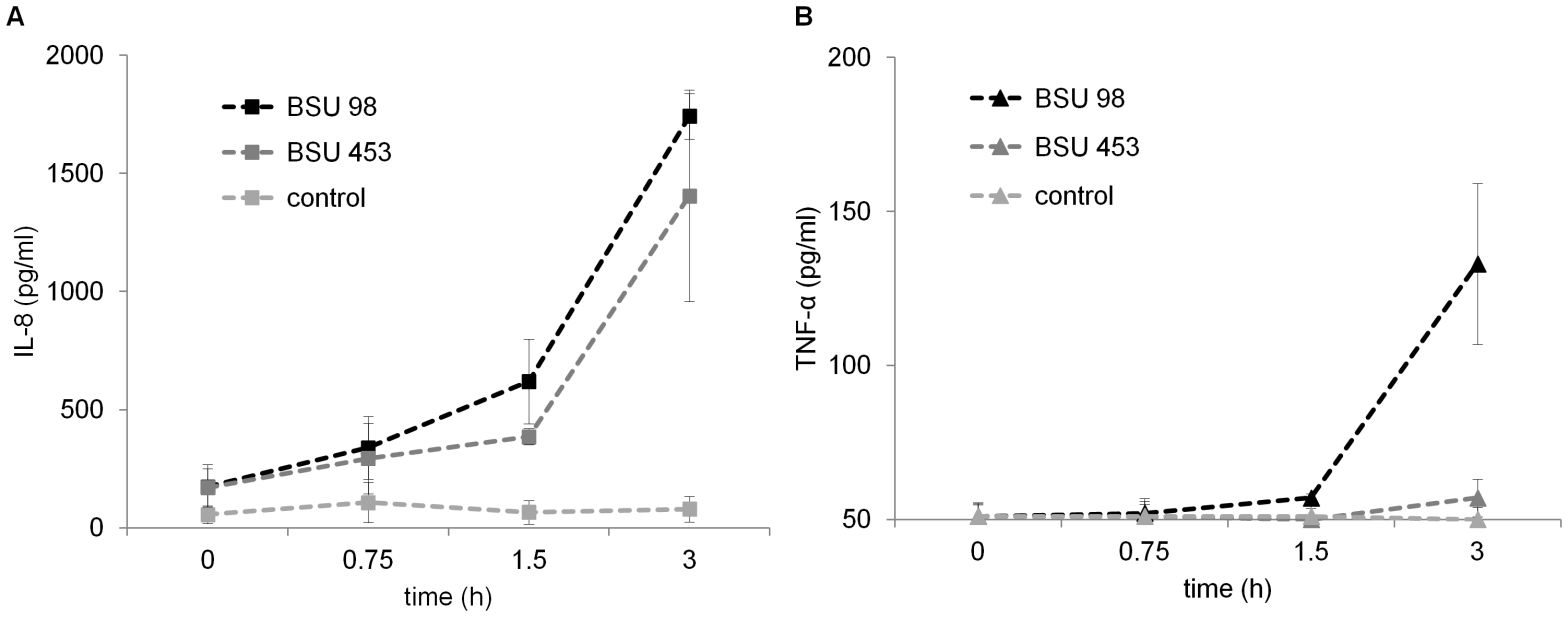
Intracellular *S. agalactiae* induced cytokine expression in THP-1 macrophages. THP-1 macrophages were infected with hemolytic (BSU 98) and nonhemolytic (BSU 453) GBS at a MOI of 1∶1 at indicated time points. Extracellular bacteria were killed by antibiotics for an additional 1 h. **A**) IL-8 and **B**) TNF-α levels were measured in the supernatant by ELISA. Uninfected cells served as control. Data shown are the mean ± SD of three independent experiments.

The strength and efficiency of the immune response of the host is dependent on the release of proinflammatory cytokines. We investigated the induction of TNF-α and IL-8 from *S. agalactiae* infected THP-1 macrophages. Both BSU 98 and BSU 453 induce marked production of IL-8; however there was no overall difference in the release by macrophages (Fig. 6A). Nevertheless, the production of TNF-α from the infected macrophages in response to *S. agalactiae* is delayed. However a significant difference in the ability of the two *S. agalactiae* strains to produce TNF-α was observed after 3 hours of incubation, suggesting a functional role of TNF-α in *S. agalactiae* pathogenesis (Fig. 6B).

### Induction of Proinflammatory Cytokines by *S. agalactiae* Cell Wall Preparations

*S. agalactiae* molecules located on the cell surface or secreted into the supernatant play an important role for the pathogenicity of the bacteria. Previous literature showed that the β-hemolysin contributes to the stimulation of IL-8 release [8] [16]. Because a high IL-8 release from THP-1 macrophages was also observed following stimulation with strain BSU 98 as well as the nonhemolytic strain BSU 453 (Fig. 6A), we hypothesize that the presence of β-hemolysin in BSU 98 may not be the sole mediator for IL-8 release, suggesting the involvement of other bacterial factors that could trigger the release of proinflammatory cytokines. The bacterial cell wall components of gram positive bacteria are a major proinflammatory stimulus and trigger the innate immune system similarly to lipopolysaccharide (LPS) of gram negative bacteria [15] [18]. To investigate the role of cell wall, THP-1 macrophages were stimulated with *S. agalactiae* cell wall preparations from hemolytic and nonhemolytic strains.

**Figure 7:**
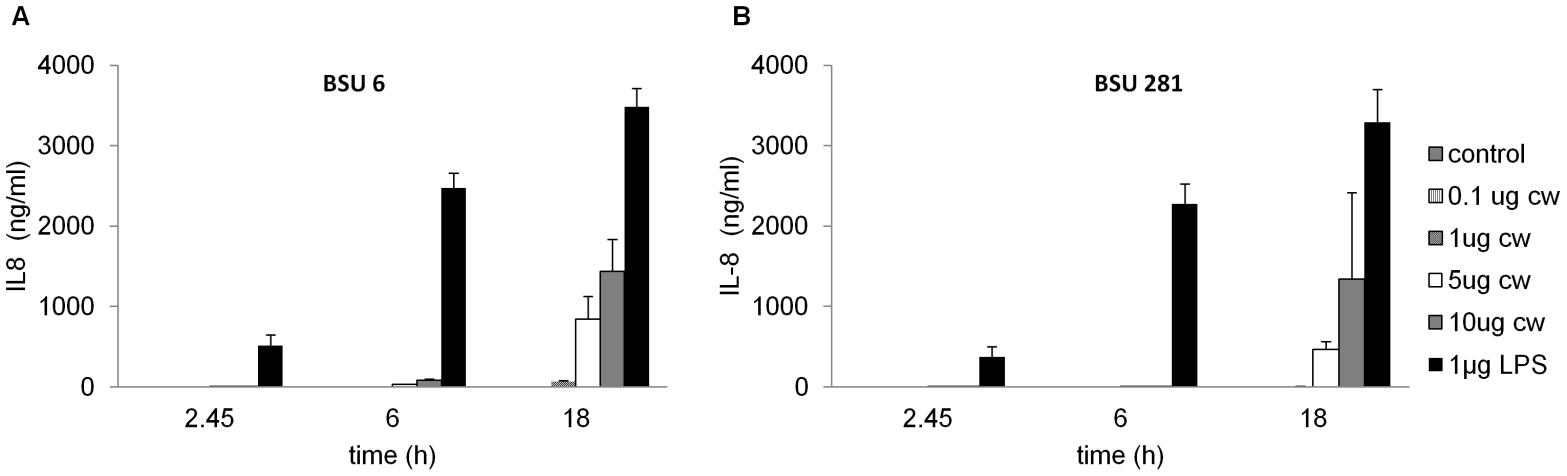
Similar IL-8 expression pattern in response to purified cell wall preparations from hemolytic and nonhemolytic *S. agalactiae* strains. 10^6^ THP-1 macrophages/well stimulated with 0.1, 1, 5 and 10 µg/ml of cell wall preparations from (**A**) BSU 6 (hemolytic) and (**B**) BSU 281 (nonhemolytic) for indicated time points. Treatment of cells with 1 µg/ml of lipopolysaccharide served as positive control, unstimulated cells in medium as negative control. Data shown are the mean ± SD of three independent experiments.

A dose and time dependency was observed in the production of IL-8 following stimulation with 0.1, 1, 5 and 10 µg/ml of cell wall preparations. Maximum stimulation was observed for 1 µg of LPS that served as a positive control in the assay. As β-hemolysin activity is lost during the cell wall isolation method, the resultant cell wall fragments from BSU 6 and BSU 281 show a similar response towards the production of IL-8 as shown in Fig. 7A and 7B.

## Discussion

Severe invasive *S. agalactiae* infections are not only a major cause of infections in newborns but also in nonpregnant adults. Bacterial invasion and disease pathogenesis is a complex process that is achieved through numerous virulence factors. The *S. agalactiae* β-hemolysin is considered as one of the most important virulence factors in this context. Invasive *S. agalactiae* infections are almost exclusively caused by β-hemolytic strains. However, the role of the *S. agalactiae* β-hemolysin in the molecular interaction with host cells is not completely understood.

Interestingly, the *cov* (or *csr*) system as a major regulator of S. *agalactiae* virulence genes suppresses β-hemolysin expression [19] [9]. A recent study by Sendi *et al*. found that a strain carrying a *cov* mutation resulting in a high hemolytic *S. agalactiae* variant with low capsule expression showed low intracellular survival in human neutrophils in contrast to the low hemolytic variant with high capsule expression [8]. To elucidate whether the observed difference is due to β-hemolysin or capsule expression, we used a serotype Ia strain and its isogenic nonhemolytic mutant to investigate the potential factors responsible for the different survival in eukaryotic host cells. The β-hemolytic *S. agalactiae* wild type strain was found in lower numbers in the intracellular compartment of THP-1 macrophages in comparison to the nonhemolytic mutant strain. With increasing incubation time (from 0.75 to 1.5 h), the number of recovered intracellular bacteria increases most probably due to additional uptake. Higher MOIs (5 and 10) and higher incubation times (>2 h) lead to enhanced lysis of the eukaryotic cells attributed to the cytolytic activity of β-hemolysin in the wild type strain and were therefore not included in the analysis. In contrast to this, the nonhemolytic strain caused damage of eukaryotic cells only at long-term incubation (24 h) which may be caused by the induction of apoptosis [20] [21]. To investigate if an improved survival of the nonhemolytic strain could also be observed in the interaction with granulocytes as described by Sendi *et al.*, the hemolytic strain and its nonhemolytic isogenic mutant were tested for survival (Fig. 1) following 2 h incubation with primary human granulocytes under sub-cytolytic conditions. In these settings, significantly higher numbers of the nonhemolytic strains were recovered which is compatible with the results of Sendi *et al.* obtained for *S. agalactiae* CovR/S mutants. While the major phenotypic difference between the two strains we tested is the loss of hemolysis in the mutant strain, we can currently not exclude the possibility that the mutation of the hemolysin transporter causes an altered expression of other virulence determinants, which may contribute to the increased intracellular persistence, we observed.

It is intriguing to see that an important virulence regulator of *S. agalactiae* suppresses the β-hemolysin expression. The clinical observation that *cov* mutation and resulting hyperhemolysis are associated with devastating fulminant invasive disease offers a possible explanation for this phenomenon [8]. *S. agalactiae* is most often a colonizing bacterial pathogen causing invasive disease mainly in neonates and immunocompromised patients. Maximal expression of virulence factors does not appear to be beneficial in all stages of the course of an infection. The improved survival within professional macrophages may provide advantages like the escape from antibody attacks or the use of these host cells in the sense of a Trojan horse, as it has been observed in other pathogens [22] [23]. A recent publication describing the increased expression of the *cov* regulator in *S. pyogenes* recovered from the intracellular environment of macrophages supports this line of argument [24]. While investigating the survival of *S. aureus agr* mutants in murine models of infection, Schwan *et al.* also suggested a possible role of hemolysin expression in providing a growth advantage in *S. aureus* mixed cultures within abscesses and wounds [25]. Littmann *et al.* investigated the role of bacteria-bound pneumolysin in the survival of *S. pneumoniae* in human dendritic cells *in vitro* [26]. Recovery of higher numbers of the pneumolysin-deficient strain from human dendritic cells as compared to the wild type strain seems to be advantageous because the hemolytic activity of pneumolysin was not found to be essential for invasive disease in their experimental setup. Using a murine model of pneumococcal bacteremia, Harvey *et al.* found an enhanced *in vivo* survival of the pneumolysin deficient strain [27]. Independent observations from both groups about the pneumolysin expression dependent survival of *S. pneumoniae* support our findings suggesting that a low or absent hemolysin activity may, for some streptococcal strains, be critical for its survival within professional phagocytes.

In our experiments, we could demonstrate that the survival of *S. agalactiae* is dependent on the uptake into THP-1 macrophages. We were able to show that entry of *S. agalactiae* into THP-1 macrophages is inhibited by Cytochalasin D in a dose dependent manner, indicating that the uptake of *S. agalactiae* occurs through phagocytosis, a mechanism that actively involves actin polymerization (Fig. 3). A similar finding was made by Valentin-Weigand *et al.* for serotype III Group B streptococci using the murine macrophage-like cell line J774 [28]. Our results also support the findings of Fettuciari *et al.* who showed in a recent publication that the *S. agalactiae* β-hemolysin activates calpain leading to a severe disturbance of the cytoskeleton and may thus prevent the uptake of *S. agalactiae* into eukaryotic cells [29].

Our fluorescence microscopy data clearly distinguishes between intracellular and extracellular bacteria. However, the current study does not address the location of bacteria within the macrophages. Previous electron micrograph and fluorescence microscopic studies on various GBS serotypes have reported the location of GBS within the membrane-bound vacuole in a variety of host-cell types, including epithelial cells [17], endothelial cells [30] [31], dendritic cells [32] and macrophages [33] [29] [34]. In *Listeria monocytogenes*, the hemolysin is important in phagosomal escape and hemolysin negative mutants are thus impaired in intracellular survival [35]. However, in a recent investigation of the subcellular localization of *S. agalactiae* within J774 macrophages, no indication for the escape of *S. agalactiae* from the phagosome was observed [33]. The vast majority of *S. agalactiae* could be observed within phagosomes and survival of *S. agalactiae* under these conditions was even found to be dependent on proper acidification of the vacuoles. It is therefore not surprising to see that in contrast to findings in *Listeria* the ß-hemolysin does not appear to be crucial for intracellular survival of *S. agalactiae.*

The strength and efficiency of the immune response of the host is dependent on the release of cytokines. Previous literature has shown that group B streptococci stimulate the release of various cytokines and chemokines from human mononuclear cells and murine macrophages [36] [37]. Antibodies against TNF-α in an experimental neonatal rat model of GBS sepsis significantly increased their survival, suggesting a contribution of TNF-α in GBS pathogenesis [38]. In line with this, our results showed the release of TNF-α by THP-1 macrophages when stimulated with *S. agalactiae* serotype Ia strains. Higher TNF-α release was observed in macrophages exposed to the hemolytic strain (BSU 98) suggesting a possible role of β-hemolysin expression in the induction of TNF-α at 3 h. The chemokine IL-8 is an important marker of bacterial infections. Previous reports described β-hemolysin as a major inducer of IL-8 [16]. However, we could not find a significant difference in the release of IL-8 by the hemolytic and nonhemolytic bacteria. Use of different *S. agalactiae* strains and cell lines could account for this difference and hence the results are only partially comparable. The wild type strain we used displays only moderate β-hemolytic activity. In addition, the higher number of nonhemolytic bacteria may have compensated for the IL-8 production and therefore a comparable release of IL-8 is observed. Since a major effect of the β-hemolysin on IL-8 release could not be observed, we investigated other proinflammatory stimuli that are present in both hemolytic and nonhemolytic strains of *S. agalactiae* and may account for the observed release of IL-8. In this regard the gram positive cell wall is a likely proinflammatory candidate.

The bacterial cell wall isolation method inactivates and removes β-hemolysin resulting in a cell wall preparation consisting of peptidoglycan and lipoteichoic acids. Previous studies have shown the effect of individual *S. agalactiae* cell wall components on the release of host-derived proinflammatory cytokines, particularly TNF-α on cord blood monocytes [18]. Compatible with these results, we found that the presence of cell wall components from both hemolytic and nonhemolytic bacteria induces similar levels of IL-8 in a concentration dependent manner.

In conclusion, we were able to show that the absence of *S. agalactiae* β-hemolysin may enable bacteria to survive in higher numbers inside professional phagocytes, an ability which may be beneficial at certain stages of *S. agalactiae* infections. This explanation is partly supported by the previous report indicating that the *S. pyogenes* gene expression profile does not remain the same at all infection stages. Two-component regulatory systems allow bacteria to adapt to changing environmental conditions. In particular, later phases of infection are potentially influenced by the CovR/S two component system in *S. pyogenes* [24]. In line with this, the *S. agalactiae* CovR/S two component system could up or down regulate the expression of its target genes in a way that proves favorable for bacterial growth inside the host [9]. While our investigation does not target the CovR/S system, it is well known that this regulator suppresses β-hemolysin expression [9]. In addition, a recent publication shows the importance of the CovR/S regulator for intracellular survival of *S. agalactiae* [33]. Our data support the hypothesis that invasive *S. agalactiae* infections represent a multifunctional process that is achieved by an intricate control and regulation of specific virulence factors.
